# Do Maladaptive Schemas Put Young People at Risk for Addiction?

**DOI:** 10.5812/ijhrba.16184

**Published:** 2014-03-15

**Authors:** Christian P. Müller

**Affiliations:** 1 Department of Psychiatry and Psychotherapy, Friedrich-Alexander-University Erlangen-Nuremberg, Schwabachanlage 6, 91054, Erlangen, Germany

**Keywords:** Behavior, Addictive, Cognition Disorders, Adolescent

*Dear Editor,*

The use of psychoactive drugs is a wide spread phenomenon which occurs in virtually all cultures of the world. Most people who consume psychoactive substances may control their consumption well over their whole life span and even draw benefits from it (1). In a minority of the users, however, controlled drug use can make a transition to drug addiction. This is a psychiatric disorder which requires treatment, or even better, prevention. Although drug use is the ‘conditio sine qua none’ of developing drug addiction, there are additional factors that contribute significantly to the risk. There are genetic (2) and epigenetic factors (3, 4), and there are environmental factors. These environmental factors may be further distinguished in passively incurred and actively developed factors (5). Passively incurred factors may be the experience of a natural disaster or a major private loss, which may then trigger the escalation of drug consumption. But there are also actively developed environmental factors. One group of them is the schemas that people form. These are patterns of beliefs and thoughts about oneself, others, and the environment, which shape how we perceive our environment, how we form memories and how we generate behavior. These schemas are acquired starting in early life. They can be helpful or maladaptive. Young et al. (6) had identified eighteen maladaptive schemas which were grouped in five areas: 1) disconnection/rejection, 2) impaired autonomy/performance, 3) impaired limits, 4) other directedness and 5) over vigilance/inhibition. These schemas are believed to foster negative experiences in life and produce a rather insufficient coping with psychological pressure. Positive schemas, in contrast, allow people to experience more positive experiences and allow for a better coping with stressful life events. Previous studies have shown that an enhanced rate of early established maladaptive schemas is found in drug users (7, 8). While genetic, epigenetic and also passively incurred environmental factors can only explain a small proportion of the addiction risk, it is currently unclear which role actively developed factors might play.

Bakhshi Bojed and Nikmanesh asked in their study whether the establishment of maladaptive schemas increases addiction potential of young people and whether these early maladaptive schemas can predict addiction potential. To address this question they investigated 260 students (159 females, 101 males) of the University of Sistan and Baluchestan in the year 2011-12. They were all young adults 19-25 years of age. All subjects were administered the Addiction Potential Scale questionnaire to evaluate current addiction potential. In order to measure early maladaptive schemas, the young schema questionnaire – short form was used. The main finding of this study was a significant positive correlation between addiction potential and all of the five areas of early maladaptive schemas (disconnection/rejection, impaired autonomy and performance, Impaired limits, other-directedness and over vigilance/inhibition). In a regression analysis the authors identified the schema of disconnection/rejection as the most influential factor, which alone determined already 0.16 of the variance in the addiction potential. Together with the schemas ‘impaired autonomy’ and ‘other-directedness’ it explained 0.19 of the variance (9). These findings clearly suggest that there is a strong relationship between early maladaptive schemas and the addiction potential in young adults. Maladaptive schemas are established early in life often by family interactions. In fact, offspring copies the schemas of parents, family members or even peers. This may explain why there is a high inheritance of the addiction risk (10), but only a small part of it appears genetically or epigenetically transmitted. As the present study shows, an increasingly big role might be played by inherited schemas, where children simply copy maladaptive schemas of their parents. Maladaptive schemas not only predict addiction as it was shown in previous studies (7, 8), but already the addiction potential. Thus, they may well suit as screening instruments to identify people at increased risk of addiction and may allow focusing prevention programs in the future. 
